# Barriers to Practicing COVID-19 Preventive Behaviors Among Migrant Workers in Qatar: A Qualitative Study During the First Wave of the Pandemic

**DOI:** 10.3389/ijph.2022.1604881

**Published:** 2022-08-04

**Authors:** Ghadir Fakhri Al-Jayyousi, Mohamed Nour, Jinan Suliman, Muna Abed Alah, Khaled Ali, Sami Abdeen, Mohammed Al-Thani, Shariq Jaffrey, Hamad Eid Al-Romaihi, Elmoubasher Farag

**Affiliations:** ^1^ Department of Public Health, College of Health Sciences, QU Health, Qatar University, Doha, Qatar; ^2^ Health Protection and Communicable Disease Control, Ministry of Public Health, Doha, Qatar; ^3^ Department of Community Medicine, Hamad Medical Corporation (HMC), Doha, Qatar

**Keywords:** risk perception, COVID-19, lifestyle, living conditions, migrant workers, Qatar

## Abstract

**Objectives:** Manual and Craft Workers (MACWs), who constitute more than 80% of the population, were identified to be a vulnerable group to COVID-19 in Qatar. The goal of this study is to identify the limitations face MACWs in Qatar towards practicing the COVID-19 preventive measures and thereby designing behavioral change strategies.

**Methods:** This is a qualitative research study in which individual interviews and focus group discussions were utilized for a deep understanding of the phenomenon from key informants. Four onlive individual interviews and four focus groups (*n* = 55) were conducted and transcribed verbatim. Inductive qualitative analysis was followed to discover the themes of the interviews. Data were analyzed using constant comparative techniques.

**Results:** Major themes elicited from the analysis revealed that the barriers to following COVID-19 preventive behaviors among migrant workers in Qatar included barriers related to knowledge and risk perception; lifestyle and habits; nature of work and living conditions, and barriers related to health communication, diversified cultures, and languages.

**Conclusion:** The findings would support constructing culturally sensitive health education messages and planning for effective health communication campaigns.

## Introduction

According to the International Labor Organization (ILO) estimates, in 2017, there were over 160 million migrant workers (MWs) globally [1]. The International Convention on Migrant Workers and its Committee in 2005 defined a MW as “a person who is to be engaged, is engaged or has been engaged in a remunerated activity in a State of which he or she is not a national.” Owing to the demand created by the flourishing oil and gas industry besides the mega infrastructure projects in Qatar ahead of the preparations to host FIFA 2022, hundreds of thousands of MWs poured into the country. They comprise 86% of the total population [2, 3]. New Ad Hoc districts were established to accommodate the MWs, who are locally known as Manual and Craft Workers (MACWs).

Generally, the population in Qatar could be classified into three main categories: 1) Qataris, 2) white-collar migrants (employees and staff) 3) the yellow collar which is composed predominantly of the single male MACWs. Current estimates suggest that single male laborers (SMLs) constitute 50% of Qatar’s population [4]. Yet, single female MWs who serve in various community businesses also exist.

MACWs regularly live in shared accommodation facilities, known as labor camps. These camps vary in their capacity, and quality of in-house services like housekeeping, laundry, entertainment, and dining halls. Some of them live together in the same room and share bathrooms and kitchens [5]. They are being transported in groups by buses or vans from their shared accommodations to their workplaces.

Accumulating evidence has shown that migrant health workers are at particular risk of communicable diseases and, to a less degree, physical illnesses [6]. A systematic review and meta-analysis conducted in Singapore showed that a significant proportion of malaria, enteric fevers, hepatitis A and E, and tuberculosis diagnosed in Singapore involve migrant workers [7]. In 2015, communicable diseases accounted for 9% of work-related mortality globally [8].

The first COVID-19 case in Qatar was identified on 28 February 2020. One week later, the first cluster of cases was detected among MACWs bringing the total cases linked to MACWs to 300 in the same month. This discovery marked the activation of the country’s pandemic response plan which focused on restricting the people’s mobility, physical distancing, infection prevention, and control precautions. Similarly, many countries including Singapore, Malaysia, Indonesia, Saudi Arabia, Bahrain, Kuwait, Oman, and the United Arab of Emirates reported COVID-19 clusters in foreign workers’ camps [4, 9].

The degree of compliance with COVID-19 mitigating measures varied across the community sectors. Qataris and the white-collar sectors, which together constitute the family sector of the population [10] can act with a large degree of autonomy in response to COVID-19 preventive practices, either individually or within their families. However, the MACWs have a limited degree of autonomy due to their nature of work and accommodation layout [11]. In fact, compared with the family sector, the chances for the virus transmission across the MACWs’ dwellings seemed to be higher [12].

Several risk factors for COVID-19 transmission are inherently linked to the MACWs settings, rendering them a primary vulnerable population. The close physical proximity of less than 1 m, no or poor use of a mask, and lack of hand hygiene were key risk factors for the virus spread in the communities [13]. MACWs’ vulnerability to COVID-19 infection is largely shaped by the type of work they do and their working conditions. These include, though are not limited to, public transportation, delivery, construction, logistics, personal care, healthcare, and cleaning services. These activities can not be done remotely and they imply close physical proximity between coworkers, leaving MACWss prone to contracting the virus [14]. Lack of adequate education, literacy, and language skills as well as cultural and social barriers are believed to have limited the MACWs chances to access useful information, making them less prepared to cope with the pandemic’s constantly changing facts [15, 16].

Other challenges include access to personal protective equipments such as face masks and items of personal hygiene. The overcrowded accommodations and the use of public transportation make it difficult for them to practice social distancing [17, 18]. Thus, it was necessary to prioritize the MACWs in terms of developing control measures including the effective behavioral change methods that are particularly tailored to their contexts. Nevertheless, fostering effective behavioral change strategies relies on needs assessment and the identification and management of barriers.

During the COVID-19 pandemic, the Ministry of Public Health (MOPH) in Qatar relied on already existing remote communication channels the authorities adopted with the MACWs to spread health messages that focus on basic preventive behaviors such as hand hygiene and cough etiquette among MACWs in different languages. For example, the Ministry of Transportation & Communication (MOTC) had a program called “Better Connection,” which was established in 2014 to improve the skills of MACWs and enhance their better access to public services [19]. Since its establishment, this program has been fundamental to conveying the messages to these target communities.

This qualitative research study, as part of the national response to COVID-19, sought to identify the limitations faced by workers in Qatar towards practicing the COVID-19 preventive measures and thereby informing communication plans and designing effective behavioral messages. The findings of this study will not only benefit the effective planning for communication strategies related to preventive measures, rather it will inform healthcare policies and plans addressing the MACWs communities.

## Methods

### Participants

This is a qualitative research study that was conducted amid the pandemic in Qatar during April and May 2020. Participants were MACWs recruited upon consultation with local experts on labor settings and the selection was performed jointly with Better Connection officers who offered a list of Champion Digitals. A purposive sampling strategy was followed to recruit these workers, which allowed the rich exploration of our research question [20].

Participants for the in-depth interviews were selected from the MACWs who are among the MOTC Digital Champions but are assigned supervision roles in their camp management. Participants consisted of three males and one female with all four of them being 30 years old. The female participant was Filipina, and the three males were Indian. To assure confidentiality, they were assigned numbers: 101, 102, etc. (See Table 1 for participants’ characteristics).

**TABLE 1:** Participants' characteristics of individual interviews (Qatar, 2020).

Participant #	Gender	Age	Education	Nationality	Job
101	Female	30	Bachelor’s degree in administration	Filipina	—
102	Male	30	A degree in engineering	Indian	Employed at an undisclosed company
103	Male	30	—	Indian	Construction worker in the Industrial Area
104	Male	30	—	Indian	Driver

We also purposefully selected the participants for the focus groups by observing the diversity of nationalities, level of education, the company’s nature work, and scale of business. While the nature of work implies the degree of interference with the community, the scale of business implies the level of resources the company possesses, influencing its capability to provide and ensure a healthy and safe environment for its staff. Members of the focus groups included male MACWs between the ages of 27 and 42 with the majority being from India, Pakistan, and Sri Lanka. They had various jobs such as camp supervisor, facility department camp administrator, inspector, accommodation administrator, facilities manager, and administrative assistant. Most of them had received higher education and held academic certificates, such as a diploma in Hotel Management, a bachelor’s degree in Mechanical Engineering, and a Master of Business Administration in Human Resources. To assure confidentiality, they were also assigned numbers: 201, 202, etc.

### Data Collection

The study’s proposal was reviewed and approved by the Ministry of Public Health (MOPH) in Qatar (IRB approval number: ERC-826-3-2020). A consent form was provided to those who agreed to participate prior to their participation. Data collection continued until we reached data saturation, which is the stage when you feel you have rich data answering your research question and you are not adding any new information, thus there is no need to interview other participants [21].

Four online, semi-structured, individual interviews and four focus groups (*n* = 55) were conducted. Around 10–14 members were recruited for each focus group. At the outset of either the focus group discussions (FGDs) or the in-depth interview sessions, the session facilitators explained the study, its purpose, how the data will be used, and the anonymity of the participants, indicating that they have the choice to quit anytime they want, and that agreeing to record the sessions will be considered as a consent.

An interview guide was developed in accordance with the purpose of this study. It was pretested by conducting four individual interviews and then modified to enhance the clarity and effectiveness of the questions in eliciting information-rich responses from the participants in the FGDs. The interview guide explored the following: 1) the participants’ knowledge of COVID-19 risk factors, symptoms, and routes of transmission, 2) risk perception, 3) preventive measures including social distancing and hygiene practices, 4) barriers/challenges that hinder participants from following prevention practices, 5) the participants’ travel history, 6) best practices for self-imposed home quarantine when experiencing symptoms, and 7) the best medium/channel to reach participants and their families and educate them on COVID-19 prevention practices.

Both the individual interviews and FGDs were conducted online between April-May 2020 using WebEx to adhere to COVID-19 safety measures. Public health and medicine students who were volunteering at the Ministry of Public Health were trained by the corresponding author for this project to conduct these interviews and FGDs. Each FGD lasted approximately 2 h and each individual interview lasted approximately 45 min. All interviews were conducted in English. Data was saved on the computers of the principal investigators with password-protected and accessible only to them. The data were treated anonymously; no names or information related to the subject’s identity were used for data analysis. The individual interviews and FGDs were audio-recorded and transcribed verbatim for analysis.

### Data Analysis

Thematic analysis was employed to elicit major themes in the data. Constant comparisons were conducted to differentiate one theme from another and to identify the dimensions of each theme (subthemes). The analysis process started after conducting and transcribing the first individual interview. Coding was the first step in the analysis in which the first transcript was coded line-by-line. A codebook was constructed with main categories, themes, and subthemes [22]. Following this, the interview guide was modified, and probing questions were added to help capture a rich understanding of the main themes in the other interviews, then the team conducted three more interviews.

Next, to ensure an acceptable degree of representation, focus group discussions were conducted with workers from different shared accommodations (workers’ camps) and different companies with varying management styles. All transcripts were coded, and new themes were added to the codebook. Inductive and constant comparative techniques were performed to look for similarities and differences between participants [23]. With each addition of new data, themes were added and modified to reflect changes from new information.

In order to enhance the credibility of our research findings, the research team followed triangulation in the data collection and analysis. For the data collection, the team used individual, semi-structured interviews, and focus group discussions. During the analysis process, the research team worked independently to analyze the data to help the verification process. Team members read through the transcripts and identified common themes separately, then the team came together to collectively discuss the results and reach a consensus regarding the themes and categories. The analysis process was finalized in July 2020.

## Results

Analysis of the individual interviews and focus group discussions revealed that there were various barriers that prevented workers from following the recommended preventive measures for COVID-19: barriers related to knowledge and risk perception, barriers related to MACWs’ lifestyle and habits, barriers related to MACWs’ living and working conditions, and barriers related to health communication, diversified cultures, and languages.

### Barriers Related to Knowledge and Risk Perception

Participants revealed that workers need to be educated about preventive public health measures and understand the routes of COVID-19 transmission to protect themselves and others. The majority of the participants in our study mentioned that most of the workers are illiterate, which might affect their comprehension of different health promotion messages on hygiene practices and social distancing. Consequently, some workers underestimated their risk of getting COVID-19 and gave little attention to health messages from the MOPH and other resources in Qatar. This may have increased the risk of COVID-19 infection, as one participant from the individual interviews explained:

“Most of them are uneducated about the virus. I can be infected anytime since I am surrounded by uneducated workers who do not understand how dangerous is the situation. If one is affected, we as workers will all be affected since we share accommodation and work sites.” (Participant #102, male, from India).

Participants explained that most of the workers were not motivated to follow the recommended guidelines for hygiene, nor eager to listen to health educators or follow their advice. On the other hand, they mentioned that some workers were scared of the disease and did not wish to discuss issues related to COVID-19 with others. The sense of fear was dominant among some workers at the first peak of the pandemic in Qatar in 2020. Another participant from the individual interviews said:

“They come from different cultures and have different languages. Workers are scared to talk about the disease. I am teaching and reminding other workers about how to use sanitizers, but they are not motivated, they do not listen, and if they do not want to listen to me, I cannot just force them.” (Participant #103, male, from India).

### Barriers Related to Manual and Craft Workers’ Lifestyle and Habits

Participants mentioned that the lifestyle of workers also had a significant impact on their behaviors. Some workers in these camps use items, such as towels, plates, cups, and other personal belongings of their friends or colleagues when they are not around. This lack of boundaries facilitates the spread of the virus among workers and worsens the situation in their camps. Additionally, participants mentioned that since most of the workers are single, they would partake in large social gatherings when they feel homesick, which increases their chances of getting infected. One participant said:

“They have some bad habits, which will spread the disease. Some workers will use your personal stuff when you are not there in the room, such as your towels and plates. And regarding social distancing, they are far from their families and country of origin. They want to socialize and spend some time together. They used to do this, and now even more than before with the COVID problem.” (FGD #1participant, male, admin assistant, from Sri Lanka).

### Barriers Related to Manual and Craft Workers’ Living and Working Conditions

Some participants mentioned that the working conditions were one of the barriers that prevented them from following the recommended hygiene practices and physical distancing guidelines. They explained that some companies did not follow basic requirements for employee health and safety. For instance, some companies did not provide soap and sanitizers at work sites. Construction workers explained how washrooms, when available, were located far away from the worksite, and many were reluctant to walk far just to wash their hands. They also mentioned that they always work in large groups and take the same bus to and from work, making it difficult to maintain social distancing. One participant who is a “camp boss” said:

“Despite proper education, I am worried about poor understanding and a lack of adherence to the rules as they are not often practical in the workplace. Workers are given gloves, a hammer, and a helmet, but they prefer to work without gloves because it is easier and faster to do their job that way. They use a hammer without gloves and then pass on the hammer to others. So, there is sharing of tools.” (FGD #2 participant, male, from Pakistan).

Workers faced similar challenges in their living settings. Participants revealed that they were sharing accommodations with a large number of workers. As one participant who is an accommodation admin mentioned:

“There is no space to maintain social distance in our bedroom; there are eight people in one accommodation, which made it hard to maintain a safe space. We are too many people gathered in one kitchen to cook our meals since there is no space in the accommodation.” (FGD #2 participant, male, from India).

On the other hand, some participants mentioned that the living and working conditions encouraged them to follow preventive measures for COVID-19. They had positive perceptions of the precautions being followed by their employers, as one participant explained: “Gates have sanitizers, accommodations have soap and tissue, and transport vehicles are disinfected by drivers daily. Companies would stress how important hand washing is.”

Another participant from focus group # 3 talked about how transportation has limited capacity to enhance social and physical distancing:

“Buses are at half strength; we stand one meter apart. All bus drivers are briefed on disinfecting the bus. We all wash our hands and are only then allowed to go into rooms. Daily sanitizing with spray for all room handles and toilet door handles and handrails. There are low chances of getting infected. We work indoors, always wear masks, use masks, always wash hands, and practice social distancing.” (FGD #3 participant, male, from India).

### Barriers Related to Health Communication, Diversified Cultures, and Languages

Participants stated that worker communities constitute people from diverse backgrounds and cultures who speak different languages, thus they emphasized the importance of considering this diversity when constructing health education messages and planning for health communication campaigns. Participants mentioned that social media would be the most effective communication channel to reach workers, considering the language inclusiveness of the posted educational materials. One participant from focus group # 4 explained, “We use all social media platforms: Facebook, Insta, Snap, and WhatsApp. They can manage the outbreak by counseling us, educating us about how to be clean, how to prevent infection, and using different languages to reach all workers.” Finally, participants suggested that the health messages shared with workers should be created in plain language for easy comprehension and to reach the largest segment of workers. These messages should be about proper handwashing techniques and physical distancing, and resources related to the latest guidelines and recommendations.

## Discussion

Our study is the first of its kind to explore limitations that the workers encountered in practicing COVID-19 preventive behaviors in Qatar. In 2020, the basic control measures set forth for COVID-19 included covering the mouth and nose, handwashing and using sanitizers, limitation of social movement, and social distance. The results of this study highlighted several challenges to putting these measures into effect among the MACWs communities.

Most of the MACWs camps in Qatar were designed to accommodate workers in groups that amount to huge numbers, making the frequent and persistent social blending inevitable [24], which was the case in other GCC countries such as Kuwait [25]. For MACWs staying at home means constant exposure in the sleeping rooms, kitchens, dining halls, laundry, corridors, and other common places like gymnasia and entertainment rooms. The overcrowded MACWs housing in Qatar was found to be one of the strong predictors and substantial contributing risk factors for developing different health problems amongst MACWs in the country [26]. Only when they report to their worksites, they will have 8–10 h in the open outdoor settings. Meanwhile, the trips to worksites and back to their accommodations, which take about an hour on average, are another form of risk exposure.

Similarly, the nature of the manual work is usually done in groups and requires the exchange of equipment and tools. SARS-CoV-2 can be contracted from both symptomatic and asymptomatic patients [27]; therefore, it is recommended to maintain social distance. SARS-CoV-2 can be transmitted through direct or indirect contact with the droplets from infected persons or inanimate surfaces (e.g., desks, tables, work gloves, bus doors). Once these droplets contaminate the hands, the virus can then enter the body through the oral and nasal cavity, the eyes, and other mucous membranes, as people habitually touch their own faces [27–29]. In work involving such scenarios, distancing and frequent cleaning of equipment would seem neither feasible nor sustainable. Using disposable plastic gloves was introduced as an alternative according to the participants in our study. However, if not coupled with careful awareness about the frequent change besides repeated hand washing, gloves might contaminate and rather be a dangerous source for spreading the infection [30].

Tackling occupational hazards by only providing a preventive tool is not enough as found in a study examining the knowledge and practices among male cement workers (*n* = 153) in the United Arab Emirates (UAE) [31]. Workers who have been provided with protective facial masks from the dust; however, with minimum to no training for the majority of the participants on how and when to use the masks, their adherence to the proper use of the personal protective equipment was negatively affected [31].

Despite the authorities have issued a number of regulations aiming to promote the safety standards for the MACWs at the worksites and accommodations, the participants indicated that following their implementation is a challenge. For instance, the Ministry of Administrative Development, Labor, and Social Affairs (ADLSA), recommended that MACWs should cover more work to reduce the population density in the workplace; accommodations should allocate six square meters per worker for their safety; and that MACWs’ buses should be limited to a maximum of 50% of its total capacity [32]. While some business owners have complied with the bus regulations, as reported by the participants, sustainability is challenging. Monitoring violations to compliance and implementing disciplinary measures are needed to ensure sustainability.

Communicating preventing guidelines and instructions is helpful, but, compliance with them is conditional on assessing their feasibility. Ottawa Principles on Health Promotion and the Integrated Marketing and Communication for Behavioral Impact (COMBI) have provided practical guidance on assessing the prerequisites for compliance with the preventive practices prior to requesting target communities to embark on practicing them [33, 34]. Consistent with the best practices stated in these guiding documents, several participants mentioned the need to provide soap, sanitizers, and masks on regular basis. However, the allocation of such resources implies an unaccounted expense for the business owners such as the cost of extra trips to comply with the safety recommendations of transportation, and the cost of renting bigger accommodations to help in maintaining social distancing.

The majority of the MACWs have limited education, a barrier that has been underscored by those interviewed in this study. Thus, workers lacked sufficient knowledge about preventive measures regarding COVID-19. Low education and literacy, and language barriers are known as factors that hinder effective communication [35]. Similar to their role in ensuring the compliance of their workers with the occupational safety requirements, supervisors can play a pivotal role in informing their MACWs about the latest developments in health and safety guidelines, reflecting gaps in resources like soap and masks, and encouraging the MACWs to continue adherence to the preventive practices. The long-term investment in education and particularly health literacy will likely provide long-term and sustained positive effects [36].

At the time of conducting this study, the Ministry of Public Health sent out messages through mass media outlets to help target populations understand the risks and comply with the prevention practices. No doubt such a remote form of communication, which has been the primary policy for sharing the disease updates and public guidance in consistence with the social distance requirement, will fall short of achieving the intended impact compared to the face-to-face forms of communication where lack of attention and other documented communication barriers, like the language and cultural barriers, will be averted [37].

An Iranian study reported that considering the culture of the target community is necessary to control an ongoing outbreak [38]. In a multicultural country like Qatar, many single workers come from different ethnic backgrounds and approach health practices differently [39]. Participants from our study acknowledged this fact and therefore recommended that any future health-related messages and campaigns should consider the diverse group of MACWs to ensure healthcare equity. Promptly responding to this issue, authorities in Qatar released several messages in six languages covering most of the target MACWs [39].

As per the participants, more effort needs to be done to effectively engage the workers’ communities. Social media was signaled as the primary channel to obtain information and guidance for the MACWs, which aligns with previous research that explored the risk factors related to diarrheal diseases from the perspective of male workers in Qatar. The study suggested communicating health education messages through using a social network, such as WhatsApp channel to improve food hygiene practices among camp workers [40].

### Recommendations to Stakeholders

In order to protect workers’ health in Qatar and other countries with this segment of the population, employers and public health professionals should collaborate to assess this population’s needs when planning and implementing health promotion and community engagement campaigns to embrace effective practices; carry out environmental assessments to determine the risks as well as the MACWs’ practices that influence the infection odds and thereby guide the health promotion interventions; effectively involve the business owners, MACWs, the competent authorities, and stakeholders in addressing the needs that are required to ease and encourage the compliance with the preventive measures at the individual and community levels; and carry out frequent evaluation studies to assess the impact of the awareness activities to enhance the implementation of COVID 19 and other public health preventive measures. Our study also demonstrated that employers need to be more aware of their workers’ health, especially given the strong correlation between migrants’ health and their productivity.

### Strengths and Limitations

To our knowledge, this study is the only one in Qatar and among a few studies from the Gulf Region addressing the barriers to practicing preventive behaviors related to COVID-19 among MCWs. The qualitative research design helped us understand the complexity of this behavior and reflected on the different contexts shaping the barriers. Another strength of this study, is the different triangulation strategies followed in the data collection and analysis, which have enhanced the credibility of the findings of this research study.

On the other hand, one of the main limitations in our study is the sampling biases resulting from the recruitment of participants by the officers in the “Better Connection” program. For example, the recruited participants might have been selected from companies that were enjoying resources and internet access better than other companies; therefore, the study sample was biased and relatively less diversified. Additionally, the interviews and FGDs were conducted via internet-based technologies, which has limited the ability to extrapolate body language and take field notes that are deemed necessary to help the interviewers elicit the alluded non-verbal signals. Although the interviews and FGDs were held virtually, these participants might have been hesitant to tell the true story thinking they might lose their jobs.

Finally, as the pandemic was in its early phase when conducting this research study, the authors would like to mention that knowledge and health literacy about COVID-19 transmission has greatly improved since the study was conducted, because of the effort the Ministry of Public Health has made toward enhancing awareness related to preventive measures among the workers’ community. Future qualitative research can be conducted to have a rich understanding of the influence of the culture of origin on shaping workers’ behavior related to preventive measures and other health behaviors. Quantitative research would also help consolidate the findings on barriers to practicing the public health preventive measures among MACWs.

### Conclusion

MACWs, as individuals and groups, faced various challenges in following COVID-19 preventive measures ranging from receiving and understanding the health messages, to lack of resources through inconducive living and work conditions. Mass media forms of awareness-raising, which constituted the only choice in the early phases of the pandemic, were insufficient to satisfy the MACWs’ need for awareness about the disease facts, developments, and preventive guidelines. Cultural sensitive messages in the MACWs’ languages are needed, and other forms of in-person communication are necessary to overcome the barriers to effective communication. MACWs’ supervisors at the workplace and accommodation can play a critical role in bridging the communication gap between health authorities and MACWs communities. The findings of this study will support planning for effective communication strategies related to preventive measures and give information regarding healthcare policies and plans addressing the MACWs communities.

The manual nature of MACWs’ work, like sharing equipment and tools, together with their accommodations’ layout, like the shared bedrooms and vehicles, made compliance with the recommended practices challenging and unsustainable. Prior to disseminating awareness messages, the regular provision of masks, soap, sanitizers, and disinfection materials, together with appropriate shared accommodations that enable practicing safe distancing is necessary to empower the MACWs towards complying with the recommended practices. Our study shed the light on the crucial responsibility of employers to protect workers who are not able to understand how to protect their health, which would prepare them to respond to other infectious disease outbreaks or pandemics.
